# Pinoresinol as a Potential c-Myc Complex Modulator: An In Silico Study

**DOI:** 10.3390/cimb48080833

**Published:** 2026-08-17

**Authors:** Arnulfo Villanueva-Castillo, Claudia Mancilla-Simbro, Fernando Villa-Diaz, Alberto Ramírez-Mata, Cesar F. Pastelín-Rojas, Ruby S. Moreno-Mejía, Hermilo Lucio-Castillo, Briseida L. Castro-Bautista, Carlos G. Castillo-Sosa, Fátima Matamoros-González, Alexis Cruz-Espinosa, Angélica Abascal-Grajales, Mónica A. Olea-Amezcua, Evili Báez Castillo, Laura G. Hernández-Aragón, Alejandra Escobar Noriega, Sandra R. Reyes Carmona, Fernando Utrera Quintana, Sagrario Lobato Huerta

**Affiliations:** 1 Cuerpo Académico de Enfermedades Emergentes, Bioinformática y Dinámica Molecular CA-BUAP-274, Facultad de Medicina Veterinaria y Zootecnia, Benemérita Universidad Autónoma de Puebla, 4 Sur 304 Centro Colony, Tecamachalco 75482, Puebla Pue., Mexico; cesar.pastelin@correo.buap.mx (C.F.P.-R.); ruby.moreno@correo.buap.mx (R.S.M.-M.); briseida.castro@correo.buap.mx (B.L.C.-B.); carlos.castillos@correo.buap.mx (C.G.C.-S.); mg224462163@alm.buap.mx (F.M.-G.); alexis.cruze@alumno.buap.mx (A.C.-E.); angelica.abascal@gmail.com (A.A.-G.); fernando.utrera@correo.buap.mx (F.U.Q.); 2 ^HybridLab.^Physiology and Molecular Biology of Excitable Cells, Instituto de Fisiología, Benemérita Universidad Autónoma de Puebla, 14 sur 6301, CU San Manuel, Puebla 72570, Puebla Pue., Mexico; 3 Centro de Estudios “Justo Sierra”, Badiraguato 80600, Sinaloa, Mexico; evili.baez@correo.buap.mx (E.B.C.); sagrariolobato@cejus.edu.mx (S.L.H.); 4 Laboratorio de Simulaciones Moleculares Computacionales, Tecnológico Nacional de México, Campus Guaymas, Carretera al Varadero Nacional, Las Playitas, Guaymas 85480, Sonora, Mexico; juan.vd@guaymas.tecnm.mx; 5 Laboratorio de la Interacción Bacteria-Planta, Centro de Investigaciones en Ciencias Microbiológicas, Instituto de Ciencias (ICUAP), Benemérita Universidad Autónoma de Puebla, Building 103J, Av. San Claudio S/N, San Manuel Colony, Puebla 72570, Puebla Pue., Mexico; alberto.ramirez@correo.buap.mx (A.R.-M.); sandra.reyes@correo.buap.mx (S.R.R.C.); 6 Unidad Académica Multidisciplinaria Mante, Universidad Autónoma de Tamaulipas. Blvd. Enrique Cárdenas González No. 1201 Pte. Ciudad Mante, El Mante 89840, Tamaulipas, Mexico; h_lucio2000@yahoo.com; 7 Facultad de Artes Plásticas y Audiovisuales, Benemérita Universidad Autónoma de Puebla, Vía Atlixcáyotl No. 2499, Complejo Cultural Universitario, Reserva Territorial Atlixcáyotl Colony, Puebla 72810, Puebla Pue., Mexico; monica.olea@correo.buap.mx; 8 Facultad de Ingeniería, Benemérita Universidad Autónoma de Puebla, Boulevard Valsequillo y Avenida San Claudio, s/n, Cubo 1 Edificio ING 5, Ciudad Universitaria, San Manuel Colony, Puebla 72570, Puebla Pue., Mexico; 9 Lab de Cáncer y Comunicación Intercelular, Instituto de Fisiología, Benemérita Universidad Autónoma de Puebla, 14 sur 6301 CU San Manuel, Colony Los Volcanes, Puebla 72570, Puebla Pue., Mexico; laura.hdzaragon@correo.buap.mx; 10 Academia de Fisiología, Facultad de Medicina-, Benemérita Universidad Autónoma de Puebla, Calle 13 Sur 2706, Centro Colony, Puebla 72410, Puebla Pue., Mexico; alejandrad.escobar@correo.buap.mx; 11 Departamento de Investigación en Salud, Servicios de Salud del Estado de Puebla, 603 North 6th Street, Centro Colony, Puebla 72000, Puebla Pue., Mexico

**Keywords:** c-Myc oncoprotein, pinoresinol, molecular docking, AMBER analysis

## Abstract

The c-Myc oncoprotein is a central regulator of oncogenic transcriptional programs that remains challenging to inhibit directly, necessitating strategies that target c-Myc–associated protein complexes rather than the protein alone. This study conducted an in silico evaluation of the natural biphenolic lignan pinoresinol as a potential modulator of the c-Myc–TBP–TAF1 (TATA-binding protein (TBP)- Multiple direct interactions of TBP with the MYC oncoprotein) transcriptional complex (PDB ID: 6E16). Prior to molecular docking, the protein structure was subjected to energy minimization using the AMBER ff14SB force field to optimize conformational stability and structural reliability; ligand preparation and docking were performed with standard, widely used tools (e.g., AutoDock Vina v1.1.2; visualization and interface analyses in UCSF Chimera/ChimeraX and SeamDock). Molecular docking and binding-interface analyses identified reproducible interactions within defined pockets P_0_, P_1_, and P_2_, with pocket P_0_ exhibiting the highest Drug Score of 0.82. Binding affinities ranged from −5.0 to −7.1 kcal/mol, which are consistent with moderate docking scores typical for small natural ligands. Across multiple ligand poses, LYS327, LYS310, and ASP209 emerged as consistent interaction hotspots, with LYS327 showing the most frequent contacts. Furthermore, in silico ADMET analysis predicted a high probability of cytotoxic inactivity (0.98), suggesting a favorable safety profile compared to traditional agents like vincristine. These results support a protein–protein interface-oriented approach and position pinoresinol as a promising lead scaffold for disrupting c-Myc–associated transcriptional regulation.

## 1. Introduction

The oncoprotein c-Myc (Myc) is one of the most attractive cancer targets because it is deregulated in most tumors and its inhibition strongly affects tumor growth and survival [1,2]. However, deregulated Myc facilitates cancer formation and progression via multiple mechanisms, including tumor cell intrinsic mechanisms and lack of enzymatic activity; meanwhile, as it is expressed in normal cells, it is difficult to develop suitable pharmaceutical inhibitors [3]. MYC contributes to more than 50% of human cancers by functioning as a major regulator of transcription, controlling the expression of between 10% and 15% of genes [4]. Although most genes regulated by MYC are transcribed by RNA polymerase II, its precise role in the initiation of RNA polymerase II-regulated gene transcription is unknown [5]. Recent studies have begun overturning these situations, allowing Myc to be seen in the broader global gene regulatory control context. Moreover, Myc and its obligatory heterodimeric partner Max are essential for the coordinated recruitment and post-translational modification of the core transcriptional machinery components. Furthermore, Myc overexpression reprograms several important cellular functions and alters cellular sensitivity, driving its inhibition. Therefore, new pharmaceutical approaches include small molecules that either directly disrupt key Myc-Max interactions or indirectly interfere with Myc-directed post-translational modifications, essential for transcription initiation. The focus of these strategies is targeting critical pathways for Myc-dependent metabolism [1].

On the other hand, the transcription factor c-MYC is a potent oncoprotein. However, the mechanisms of transcriptional regulation through MYC protein interactions remain poorly understood [6]. Meanwhile, TATA-binding protein (TBP) is an indispensable component of the TFIID transcription initiation complex and is required for gene expression. The crystal structure of a 2.4 Å solution shows that amino acids 98–111 of human MYC interact with TBP in the presence of the amino-terminal domain 1 of TBP-associated factor 1 (TAF1 TAND1). It has been shown that amino acids 115–124 of MYC also interact with TBP independently of TAF1 and TAND1. Modeling revealed that this MYC region is like the TBP anchor motif found in factors that regulate TBP promoter loading. Site-specific MYC mutants that abrogate the MYC-TBP interaction impair MYC activity. It was proposed that the MYC-TBP interaction propagates transcription by regulating the energy landscape of transcription initiation complex assembly [7]. The clinical translation of MYC inhibitors has remained an arduous and intricate process, primarily constrained by the protein’s intrinsic structural disorder and its indispensable role in fundamental cellular homeostatic programs. These challenges underscore the necessity for innovative therapeutic strategies that target the interaction interfaces of MYC-associated protein complexes rather than the monomeric protein itself [8]. Recent breakthroughs in structural biology have begun to dismantle the paradigm of MYC as an ‘undruggable’ target. By elucidating actionable molecular features across the MYC oncoprotein family, researchers are now identifying strategic opportunities to disrupt critical interaction interfaces and their associated transcriptional complexes [9].

The c-Myc oncoprotein interacts with the binding of several cellular proteins through two known binding motifs, namely, the LXXLL acidic motif (e.g., E6-AP, p53), PDZ domain (e.g., hScrib, hDlg, MAGI–1 (–2, –3), MUPP1, PATJ, and PTPN3), and a yet unknown E6-binding motif (e.g., FADD, Gps2, hADA3, and Procaspase). The c-Myc oncoprotein mainly stimulates the destruction of many key regulatory proteins of the host cell, such as E6AP and p53, which could lead to cancer [10]. The damaged cells do not enter the cell proliferation cycle; the rate of p53 increases rapidly, inducing apoptosis. When the content of p53 decreases after degradation by the E6 oncoprotein of host cells, they are normally unable to repair DNA damage, which ultimately drives them into malignancy. New studies have shown that cells infected with the E6 protein of hr-HPV use c-Myc and interact directly with hTERT, leading to an increased phosphorylation of RNA Pol II and inducing histone epigenetic modifications of the hTERT promoter. In addition to transcriptional deregulation, HPV oncoproteins have been shown to drive profound metabolic reprogramming in cervical cancer, contributing to tumor progression and therapeutic vulnerability [11]. Moreover, triggering activation of the hTERT promoter raises telomerase activity [12]. Increased expression of hTERT in most human cancers, including HPV-positive cervical cancer, prevents telomere length shortening, leading to cell immortalization [13].

An example is the transmissible venereal tumor (TVT), also known as infectious sarcoma, granuloma venereum, transmissible lymphosarcoma, or Sticker’s tumor [14]. TVT in dogs illustrates how c-Myc plays an important role in tumor development, as it is a benign reticuloendothelial tumor commonly found in dogs in tropical regions. TVT is sexually transmitted through the exfoliation and transplantation of neoplastic cells during mating or physical contact. A unique TVT molecular feature involves the rearrangement of the c-Myc gene, which serves as a diagnostic marker. This rearrangement is derived from inserting a long interspersed nuclear element (LINE) or long interspersed repeat DNA element [15]. The insertion of the LINE-1 transposable element in the MYC gene has been reported so far in all retroposon elements [16], showing 98% homology to canine LINE-1. Therefore, the presence of this LINE near c-Myc (LINE-c-Myc) has been employed as a diagnostic tool to make a definitive diagnosis of CTVT cases using in situ polymerase chain reaction (PCR) and Conventional PCR in controversial cases [17]. Chemotherapy, particularly with vincristine sulfate, has been effective in treating TVT, resulting in remission in over 90% of cases. However, vincristine can cause side effects like myelosuppression and gastrointestinal issues. Additionally, natural compounds such as pinoresinol, a lignan found in various plant foods, have shown potential anticancer properties, exhibiting selective antineoplastic effects against cancer-derived cells while preserving healthy cells [18]. Pinoresinol induces apoptosis in cancer cells by affecting both the mitochondrial and extrinsic pathways, involving Bcl-2 family proteins and caspase-8 activation [19,20].

In this way, the aim of this study was to use a natural-origin compound, such as pinoresinol, for molecular docking calculations to investigate the molecular interaction of possible plant-origin inhibitors with the binding environment of c-Myc-TBP-TAF1 (6E16) regions. Understanding these interactions could provide new opportunities for targeting Myc activity at the transcriptional level. Resources like the Protein Data Bank (PDB), PubChem, and ADMET analysis were employed to visualize protein structures and assess the druggability of potential compounds.

## 2. Materials and Methods

To investigate the molecular interaction between the biphenolic lignan pinoresinol and the oncoprotein c-Myc-TBP-TAF1 complex, a multi-step computational workflow was implemented:

Selection of software and databases. Several bioinformatic tools and databases were utilized to perform the molecular docking analysis. Online molecular docking was executed using the SeamDock platform (SeamDock v2.0). Subsequently, the docking simulations and structural results were visualized and analyzed employing Avogadro 1.2.0 [21] and UCSF Chimera 1.16.

Preparation of Ligand and Protein Structures. The experimentally determined structure of the c-Myc–TBP–TAF1 complex (PDB ID: 6E16, resolution: 2.40 Å) was retrieved for computational analyses. Prior to docking, the protein structure was subjected to energy minimization using the AMBER ff14SB force field to ensure conformational stabilization, optimize hydrogen placement, and resolve steric clashes, thereby improving structural reliability. Concurrently, the three-dimensional structure of pinoresinol was optimized. Molecular docking simulations were then performed to predict the binding interactions, characterize the target binding pockets, and calculate binding affinities between pinoresinol and the c-Myc-TBP-TAF1 complex.

Analysis of docking results. The results of the molecular docking simulation were analyzed to understand the binding interactions between Pinoresinol and the oncoproteins. Docking simulation results were evaluated based on their binding modes, binding affinity calculations, and the identification of key amino acid residues involved in the interaction [22].

Toxicity in silico analysis. Prior to molecular docking, ADMET analysis was conducted to assess the toxicity profile of pinoresinol. To determine its toxicity endpoints, such as hepatotoxicity, carcinogenicity, immunotoxicity, mutagenicity, and cytotoxicity, as well as drug-likeness, the web servers were used. ProTox-II (https://tox-new.charite.de/ (accessed on 28 January 2025)) [23,24,25,26,27,28,29,30,31,32,33,34] and Molinspiration [22] (https://www.molinspiration.com/ (accessed on 28 January 2025)) were utilized, respectively.

To conduct molecular docking with the tumor proteins and the Pinoresinol ligand, the crystal structure of the TVT c-Myc-TBP-TAF1 protein complex (PDB ID: 6E16), with a molecular weight of 28.51 kDa, was retrieved from the PDB database (https://www.rcsb.org/structure/6E16 (accessed on 28 January 2025)). The 3D structure of the pinoresinol compound (CID: 73399) was obtained from PubChem (https://pubchem.ncbi.nlm.nih.gov/ (accessed on 28 January 2025)).

In silico: Bioactivity Assessment and Ligand Optimization. The pinoresinol bioactivity was evaluated employing tools such as Druglikeness to assess its potential as a drug candidate. This assessment included the prediction of ADMET characteristics: absorption, distribution, metabolism, excretion, and toxicity, including oral toxicity. Pinoresinol (C_20_H_22_O_6_, molecular weight 358.4) 2D structure was converted into 3D and saved in mol2 format. To ensure stable coupling with the target protein, energy minimization was conducted using Avogadro^®^ software (version 1.2.0), via the MMFF94 force field with the steepest descent algorithm, employing 4 steps per update to minimize its energy (Δ*E* = 0), to ensure stable coupling with the protein molecule. The 2D structures of the compounds were converted into 3D for docking preparation.

Binding Interface and Structural Analyses. Interactions were calculated using LigPlot [5], and the resulting 2D diagrams were verified and visualized using the Discovery Studio 2D viewer [23,24,25,26,27,28]. Additionally, 3D structural analyses and buried surface area calculations at the binding interface were performed using Chimera X software (version 1.11) [24].

Molecular docking Simulations. AutoDock Vina, via UCSF Chimera^®^ 1.16 [25], was used for molecular docking. Protein preparation involved the removal of solvent molecules, and hydrogen atoms were added using H-Bonds (histidine). Partial charges were assigned to the protein using the AMBER ff14SB force field [26,28].

Data Analysis and Modeling. Results from the molecular docking simulations, toxicity predictions, and bioactivity assessment were analyzed to determine the efficacy of Pinoresinol as an inhibitor of the c-Myc-TBP-TAF1 oncoprotein complex. To visually model the data, MATLAB^®^ (v9.14) R2023a^®^ [27,28] was utilized to perform polynomial curve fittings optimized by root-mean-square error (RMSE) values. Additionally, the SeamDock server (https://bioserv.rpbs.univ-paris-diderot.fr/services/SeamDock/ (accessed on 28 January 2025)) was employed to map the binding interface, identify hydrophobic interactions, and discriminate between weak and strong hydrogen bonds to screen for ligand conformations with optimal biological relevance.

## 3. Results

**Figure 1:**
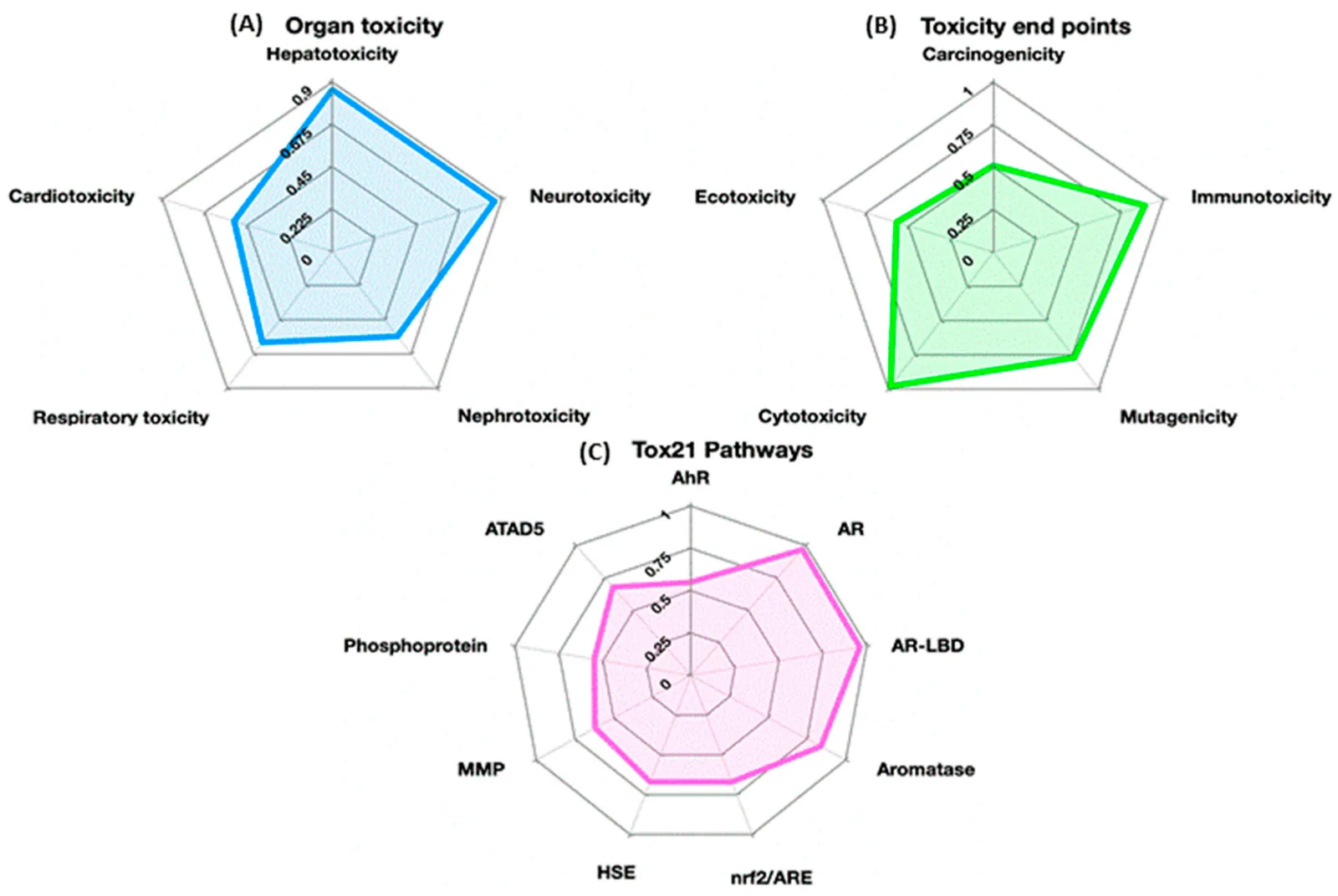
In silico toxicity probability profile of pinoresinol predicted using ProTox-II (https://tox-new.charite.de/ (accessed on 28 January 2025)): “(**A**)” Organ Toxicity: Evaluates potential toxic effects on specific organ systems, including Hepatotoxicity, Neurotoxicity, Ecotoxicity, Nephrotoxicity, Respiratory toxicity, and Cardiotoxicity. “(**B**)” Toxicity Endpoints: Assesses general toxicity endpoints, including Carcinogenicity, Immunotoxicity, Mutagenicity, and Cytotoxicity. “(**C**)” Tox21 Pathways: Measures activity across 12 stress response and nuclear receptor signaling pathways (such as AhR, AR, AR-LBD, Aromatase, nrf2/ARE, HSE, MMP, Phosphoprotein, and ATAD5). In (**A**), the label for “Neurotoxicity” is positioned closely between the radar plots of Panel (**A**) and Panel (**B**). Similarly, “Ecotoxicity” sits near the boundary (Table 1).

According to ProTox-II, pinoresinol showed a probability of cytotoxic inactivity (0.98), along with predictions of inactive hepatotoxicity (0.86), inactive carcinogenicity (0.56), inactive mutagenicity (0.77), and active immunotoxicity (0.89). The immunotoxicity was predicted as Active (0.89); this is an in silico classification that requires experimental validation and does not imply clinical toxicity (Table 1 and Figure 1).

**Figure 2:**
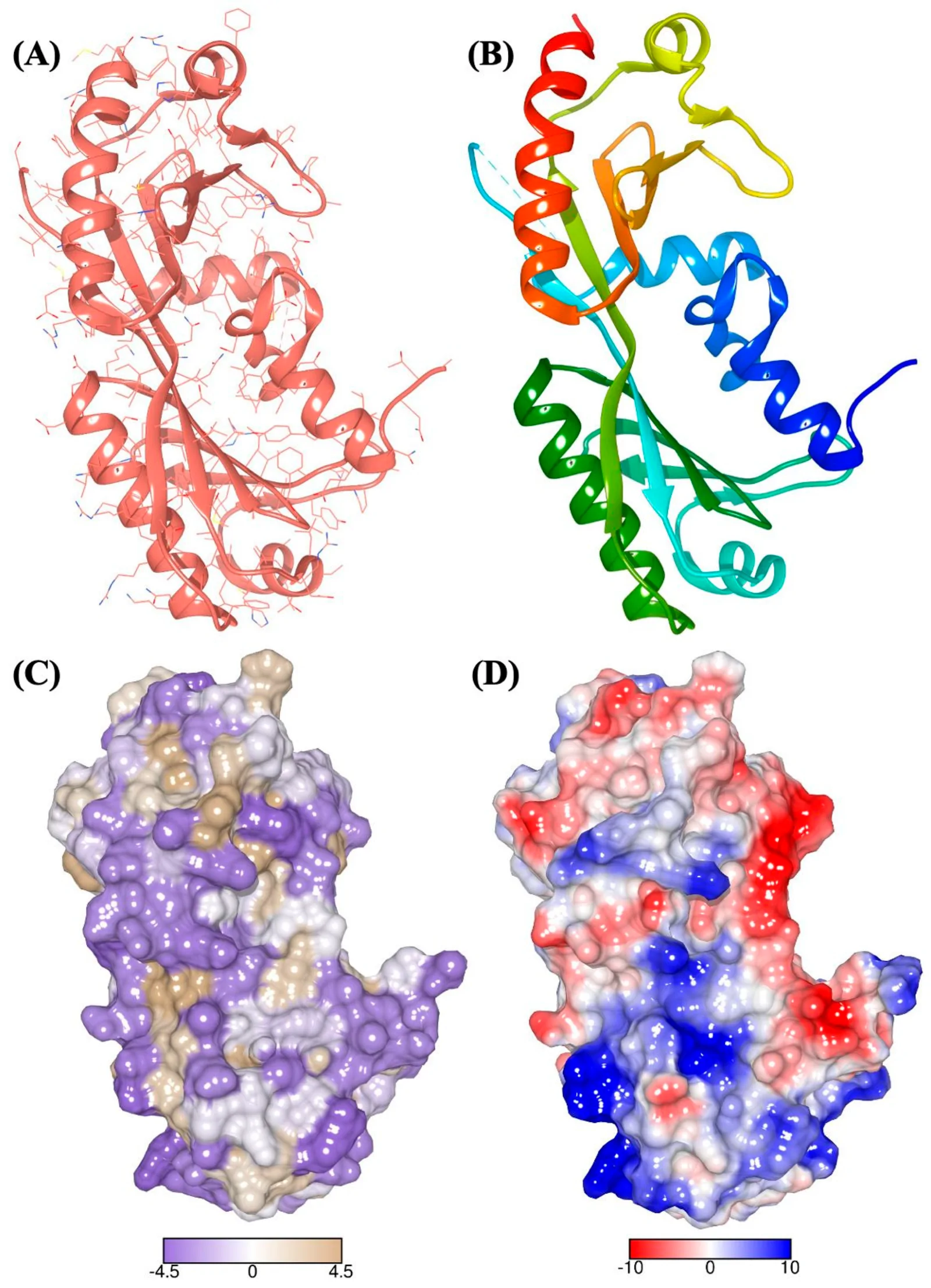
The atomic resolution structure of the c-Myc-TBP-TAF1. Cartoon representation showing the backbone in ribbon: “(**A**)” R chains of residues, “(**B**)” in rainbow, “(**C**)” solvent-excluded molecular surface (SEMS) by amino acid hydrophobicity on the Kyte-Doolittle scale and “(**D**)” SEMS by Coulombic colored surface electrostatic potential. Color code: amino acid atoms: N (blue), O (red), S (yellow), C (salmon); SEMS by hydrophobicity: polar surfaces (medium purple), non-polar surfaces (tan); SEMS by surface electrostatic potential (kcal/(mol·e)): electropositive values (blue), electronegative values (red). PDB Code: 6E16 [X1], visualization achieved by UCSF Chimera^®^ 1.16 [X2].

The biochemical model depicts the structural arrangement of the protein complex (Figure 2), highlighting the regions where potential interactions with ligands, such as pinoresinol, may occur. Understanding the three-dimensional arrangement of the protein components is essential for predicting how pinoresinol could interact with specific binding sites on the protein surface. The pinoresinol molecular structure (C_20_H_22_O_6_, CID: 733399) is shown, including its atomic and functional group arrangements, to evaluate its binding affinity and inhibitory effects on c-Myc.

**Figure 3:**
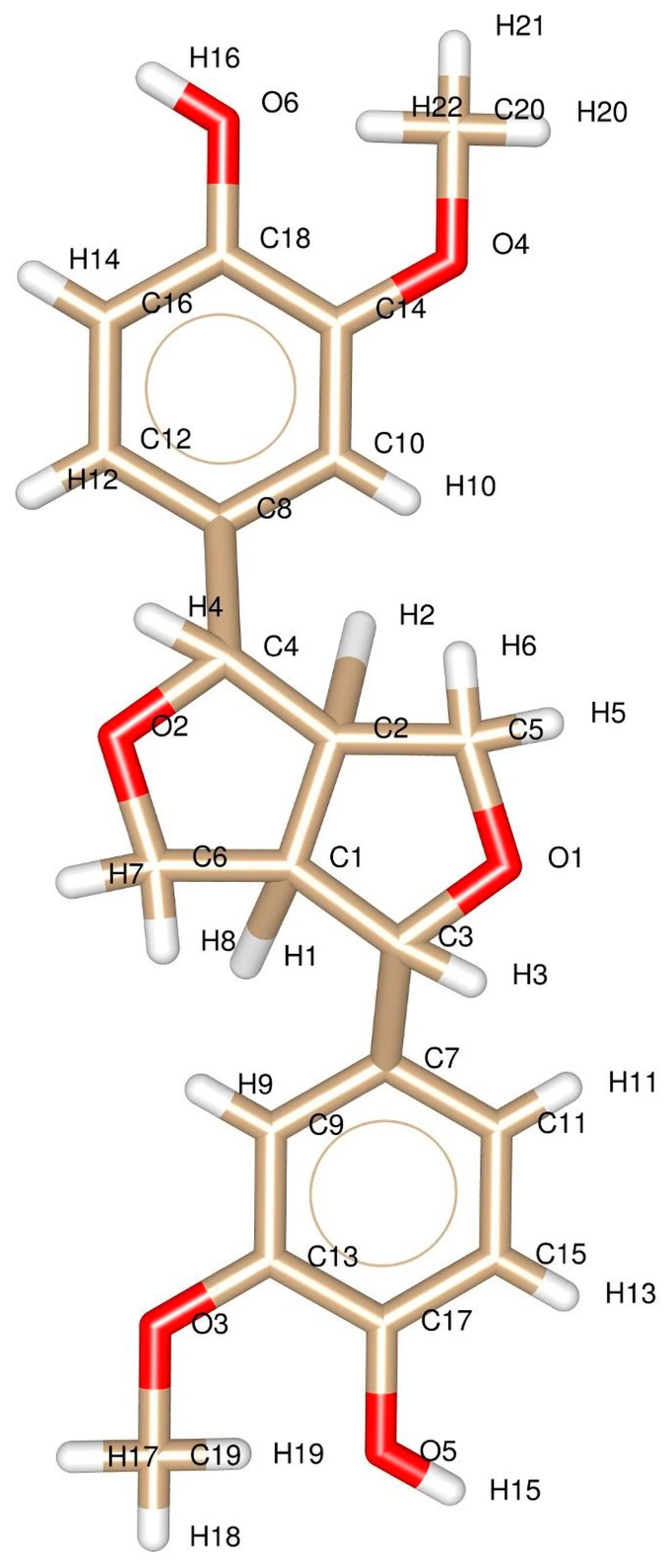
Chemical structure of (+)-Pinoresinol. The 3D structure is numbered by atom. Color code: H (white), O (red), C (khaki). PubChem Substance ID (SID): 73399. IUPAC Name: 4-[(3S,3aR,6S,6aR)-6-(4-hydroxy-3-methoxyphenyl)-1,3,3a,4,6,6a-hexahydrofuro [3,4-c]furan-3-yl]-2-methoxyphenol. Visualization achieved by: ChemSketch (version 2023, Advanced Chemistry Development, Inc., Toronto, Ontario, Canada (ACD/Labs), Toronto, ON, Canada, https://www.acdlabs.com/products/chemsketch/ (accessed on 30 January 2025)) and UCSF Chimera^®^ 1.16 [25], respectively.

Structural analysis insights into the interaction between the target protein complex (c-Myc-TBP-TAF1) and the ligand (pinoresinol), the protein complex and the ligand structure, potential binding sites and the feasibility of interactions between pinoresinol and c-Myc-TBP-TAF1 can be recognized to enhance its potential for c-Myc inhibition (Figure 3, Table 2).

Solvent accessible surface area (SASA) calculations of the molecules with a water probe radius of 1.4 Å and a 2.0 vertex density were achieved with the VEGA ZZ 3.2.2 suite (Tables S2–S4) [29]. Prior to simulation, the 2D structures of the ligand were converted to 3D for geometry optimization, and the protein structure was prepared by removing water molecules and adding hydrogen atoms. Docking parameters and configuration settings were defined according to Table 2 and Table 3 (including box dimensions, force field, charge assignment). The docking simulations were then executed via docking software (AutoDock Vina) to predict the ligand affinity of the ligand toward the protein target. Finally, the binding sites, interaction profiles, and binding energy of the ligand-protein complex were evaluated to understand the inhibitory mechanisms of pinoresinol on the c-Myc protein (Tables S5–S7).

In the context of molecular docking, Root Mean Square Error (RMSE) values were used to assess the accuracy of the docking predictions (Table 4). Lower RMSE values indicate a better fit between the predicted and experimental data. Conversely, elevated RMSE values suggest discrepancies between the predicted and experimental data, highlighting potential limitations in the docking simulation.

**Figure 4:**
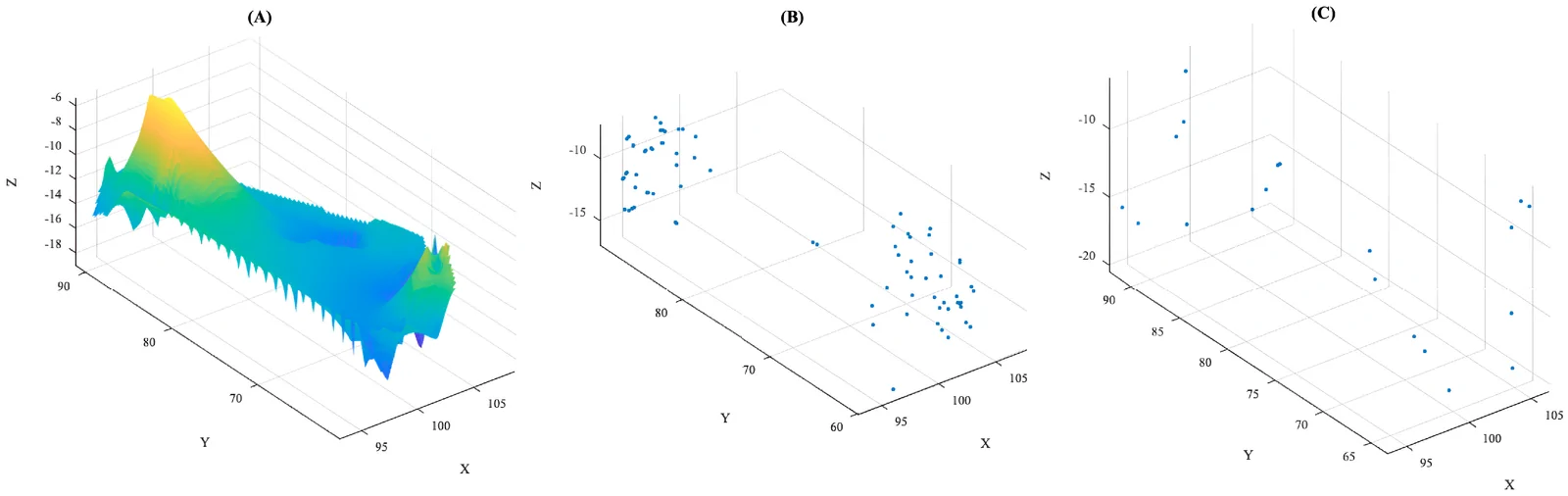
This figure illustrates the conformational clustering and structural deviation profile derived from AutoDock Vina molecular docking simulations for the interaction with the c-Myc-TBP-TAF1 complex. It is divided into three distinct panels that map energetic landscapes, spatial clustering, and structural limits: “(**A**)” Binding Energy Landscape: A three-dimensional contour plot modeled via MATLAB, illustrating the energetic distribution across spatial coordinates (X) and (Y) versus binding energy values (Z). The continuous color gradient (from blue to yellow) maps stability and energy minima across the conformational space. “(**B**)” Secondary Cluster Based on Geometric Threshold: A 3D scatter plot highlighting clustered docking conformations grouped according to a specific geometric RMSD or spatial threshold, demonstrating distinct populations of alternative binding poses. “(**C**)” High Deviation Poses: A 3D scatter representation emphasizing structurally divergent or outlier poses that mark the outer limits and structural boundaries within the c-Myc-TBP-TAF1 binding pocket.

The Discovery Studio 2D [30] analysis results (Figure 4) are crucial for understanding the interactions between ligands and receptors. The number of executions is established by the exhaustiveness parameter, which was set to 10, and influences the molecular docking simulations’ thoroughness, affecting the results’ accuracy. The AMBER analysis identified 4 nodes: OA (oxygen-hydrogen bond acceptor), A (aromatic ring), C (aliphatic carbon), and NA (nitrogen-hydrogen bond acceptor). These nodes represent specific features or characteristics of the ligands and receptors that play an important role in binding interactions. The identified nodes are within the predicted range, indicating that the molecular docking simulations align with the expected outcomes. Each node identified by the AMBER analysis corresponds to a specific property or functional group that alters ligand-receptor interactions. Understanding the significance of each node can provide insights into the molecular recognition and binding mechanisms involved. This also provides valuable information for drug discovery efforts, guiding the design of novel inhibitors with enhanced binding affinity and specificity.

**Figure 5:**
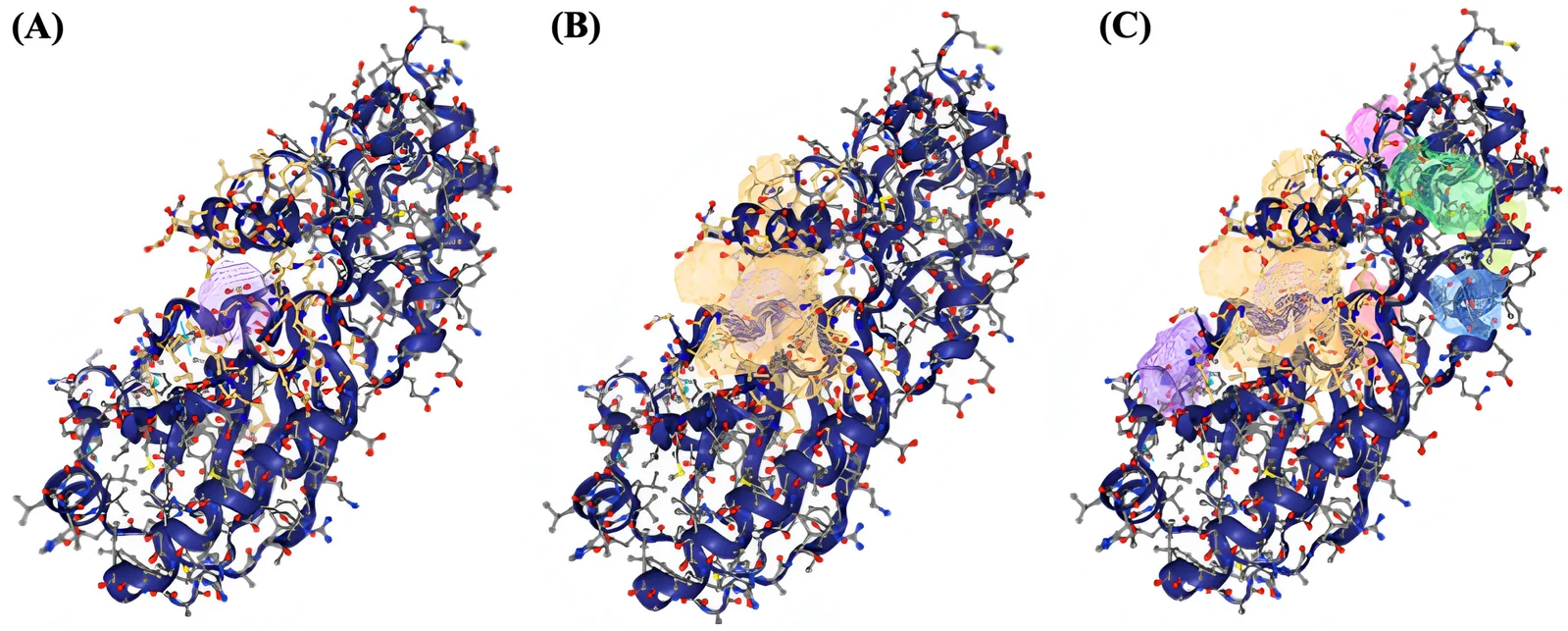
Druggable pocket selection and color-code mapping of the c-Myc-TBP-TAF1 Complex. “(**A**)” Site with pharmacological opportunity. “(**B**)” P_0: site with the highest biological interaction (DoGSiteScorer: 0.82 Drug Score). “(**C**)” P_0 (yellow); P_1 (purple); P_2 (green); P_3 (pink); P_4 (light blue); P_5 (lemon green); P_6 (hot pink). The transparent regions represent the identified pockets. The blue ribbons depict the backbone in a ribbon cartoon representation of the c-Myc-TBP-TAF1 protein structure. Atomic Color Code and Form: atoms are visualized using sticks and spheres with standard color coding: carbon (gray), nitrogen (blue), and oxygen (red). The figure highlights the identified binding sites (P_0 to P_6) (Table 6), with P_0 being the site with the highest biological interaction (DoGSiteScorer: 0.82 Drug Score), as defined in Tables S8–S10.

Pocket binding analyses (Table 5) identified seven potential pockets (P_0 to P_6). The Drug Score was utilized as a critical parameter for evaluating the binding site druggability, where higher score values indicate a greater probability of effective interaction with drug-like molecules. Pocket P_0 displayed the highest Drug Score (0.82), representing the most viable pharmacological site. Furthermore, considering the ligand’s physicochemical properties, a compound that possesses an electronegative Solvent Accessible Surface (SAS) is expected to display poor affinity for non-polar cavities, where aliphatic characteristics predominate (Tables S6–S8). This correlation is vital for interpreting the suitability of the identified pockets for the pinoresinol compound (Figure 5).

**Table 6:** Binding site detection (DogSite Score-Protein-Plus).

Number	Center			Size				Surface	Drug Score	Simple Score
	X	Y	Z	X	Y	Z	Volume			
P_0	113.1	69.5	−7.6	23.2	17.8	25.6	965.5	1079.4	0.8	0.6
P_1	109.8	79.3	−18.7	5.2	7.3	4.5	225.0	457.0	0.5	0.1
P_2	90.0	84.9	−30.6	7.0	8.1	9.3	189.3	419.6	0.3	0.1
P_3	110.2	89.0	2.1	11.1	9.5	4.9	171.0	242.4	0.3	0.0
P_4	90.1	84.3	−10.4	4.5	5.3	9.9	128.5	209.8	0.3	0.1
P_5	94.8	67.4	0.4	6.3	9.6	7.7	126.0	275.5	0.2	0.1
P_6	95.2	83.7	−10.5	4.5	7.6	10.3	110.9	254.0	0.2	0.0

Identifying ligands with biological potential provides information on the interactions between ligands and receptors, including hydrophobic contacts, weak and non-weak hydrogen bonds, number of ligand atoms, receptor atoms, and binding affinity. The calculated binding affinities (−5.0 to −7.1 kcal/mol) indicate a significantly favorable binding interaction, rather than a high-affinity interaction. These values are consistent with moderate docking scores typically observed for small natural ligands and should be interpreted as evidence of potential compatibility with the c-Myc–TBP–TAF1 binding pockets, pending experimental validation. Ten interactions (1 to 10) were chosen, those that bind to the interaction sites P_0, P_1, and P_2; interactions with affinities lower than −5.0 kcal/mol were discarded. Table 7 enumerates the best calculated positions and ligand conformations that bind to three interaction pockets where chemometric patterns were identified at the interfaces, and their interactions with specific receptor atoms. It includes details on hydrophobic contacts and weak and non-weak hydrogen bonds formed between the ligands and receptor atoms. The affinity values (kcal/mol) provide insights into the strength of the interactions between the ligands and receptors. Understanding these interactions is essential for rational drug design and developing novel inhibitors with therapeutic potential (Tables S8–S10).

**Figure 6:**
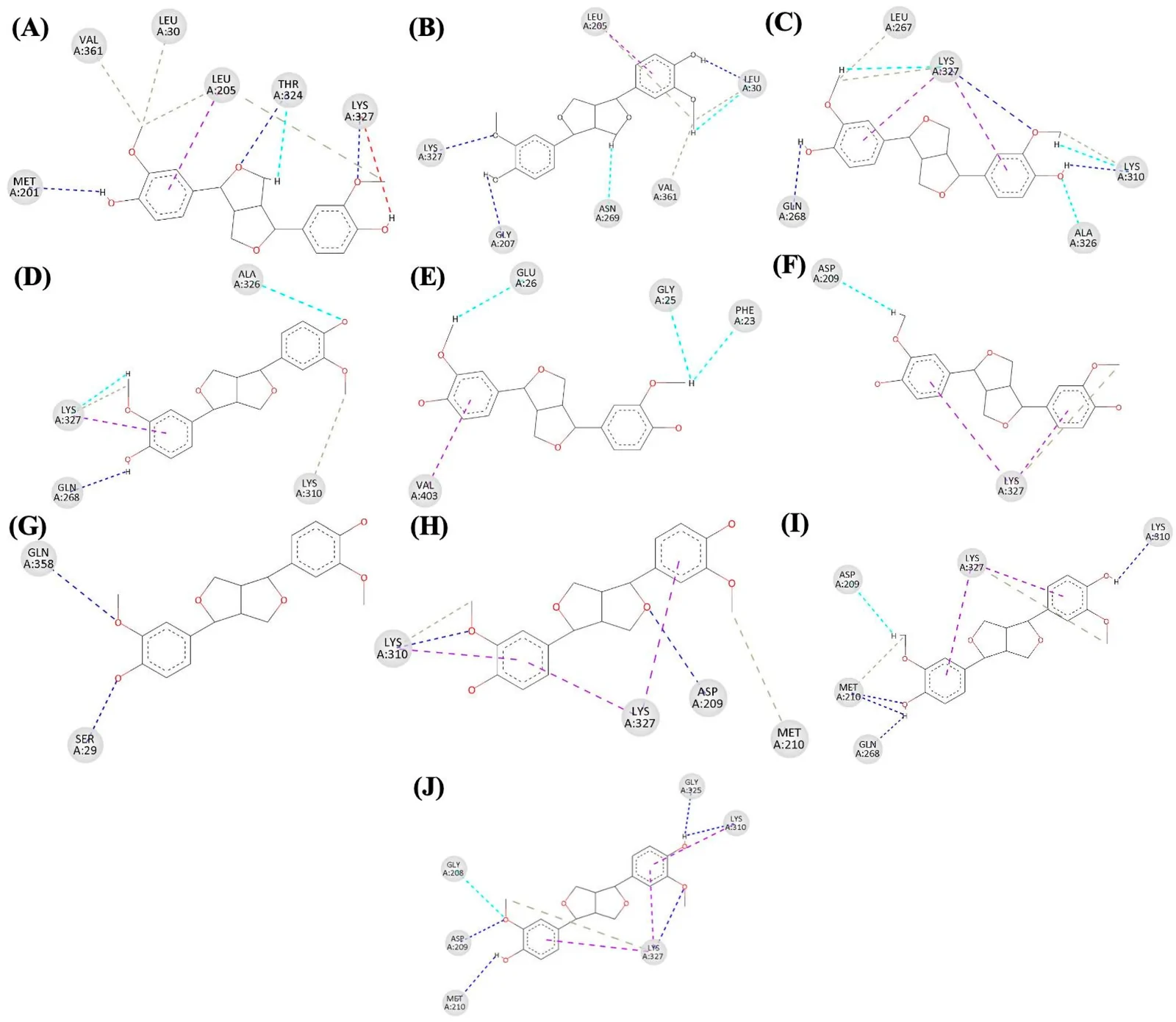
Diagrams of the different positions and conformations of the (+)-Pinoresinol complex with c-Myc-TBP-TAF1. Interactions 1 to 10 correspond to “(**A**–**J**)”, respectively. Residues are shown in light gray circles. Bonds are shown in dashed lines (conventional hydrogen bonds: navy blue; carbon-hydrogen bonds: cyan; alkyl: khaki; pi-alkyl: purple). The ligand is represented in black lines: oxygen (red), carbon (black), and hydrogen (black letters).

In detail, diagrams of the positions and conformations of pinoresinol at the interface with c-Myc-TBP-TAF1 are plotted in Figure 6, illustrating the bonds formed by the residues on the ligand. Atomic details can be found in Tables S1–S10 in the Supplementary Material. The rings of (+)-pinoresinol were classified as follows: R1 (Ring 1) comprises atoms (C8, C10, C12, C14, C16, C18), and R2 (Ring 2) comprises atoms (C7, C9, C11, C13, C15, C17) (see Figure 3). Protein-ligand interactions play a crucial role in various biological processes, including enzyme catalysis, signal transduction, and molecular recognition. These interactions are essential for understanding cellular biological functions, as well as for drug discovery and design [29].

**Figure 7:**
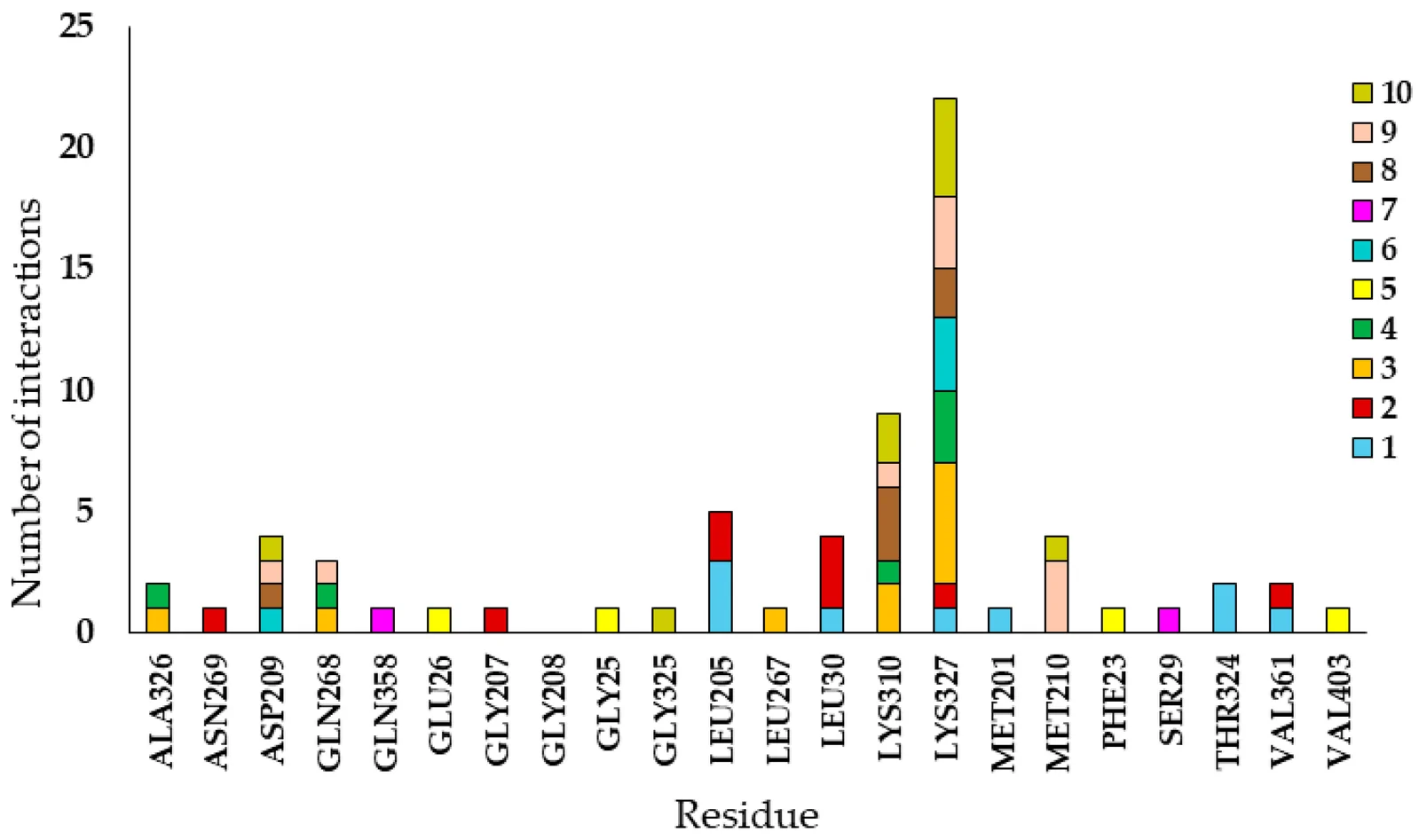
Number of interactions per residue at the interface.

The (+)-pinoresinol ligand has an approximately symmetrical structure with six oxygens (two protonated donor oxygens and four hydrogen acceptors) that could participate in conventional hydrogen bonds and two aromatic rings that are potential bond formers with π orbitals. The rest of the structure can form sigma bonds, weak hydrogen bonds, alkyl bonds, and other hydrophobic bonds. To identify potential hot spots as specific molecular targets, interaction patterns were identified on the receptor, specifically those involving residues that form non-covalent bonds with the ligand, as plotted in Figure 7. This figure shows the number of bonds per ligand interface in whole numbers. Residue LYS327 exhibits the highest number of interactions at most interfaces, except for positions and conformations 5 and 7, where it forms mostly conventional hydrogen bonds. Additionally, the second residue with the highest number of interactions is LYS310, which participates primarily in the formation of alkyl and π-alkyl bonds.

**Figure 8:**
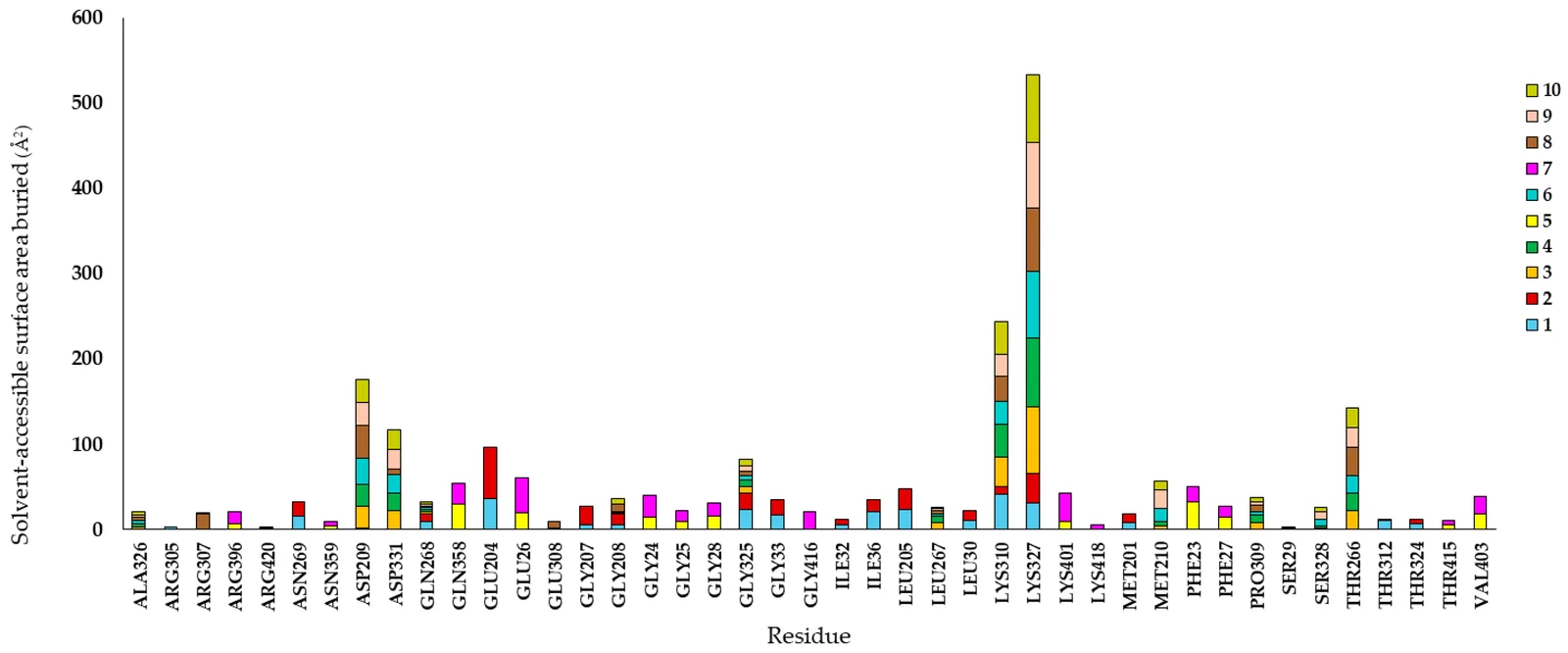
Buried surface area calculations for residue.

Molecular interactions are essential in biological processes. Non-bonding interactions between the components of a molecular complex can be modeled and determined by considering shape complementarity and physicochemical properties of the binding interface. A common way to measure shape complementarity is by calculating the surface area of a molecular complex, as well as the buried surface area. Surface area-based methods have been successfully employed in various bioinformatic areas, including the estimation of binding free energy, scoring functions for molecular docking and drug design, statistical potentials, and predicting non-covalent contacts [31]. Therefore, to complement the study and expand the search for protein hotspots, based on the atomic prediction simulated by molecular docking, the position and conformation of the ligands provide valuable information that allows the identification of interaction patterns within the protein. The buried surface area was calculated based on the position and conformation of each ligand relative to the residue, as is displayed in Figure 8 (Tables S1–S10).

## 4. Discussion

The therapeutic applications of anticancer agents, such as vincristine, have been extensively analyzed due to their cytotoxic efficacy and diverse range of actions in both experimental and clinical contexts. Vincristine has shown effectiveness in assays such as the *Drosophila* somatic mutation and recombination test (SMART), where its mechanisms lead to the loss of heterozygous marker genes in target cells. This assay’s utility extends to the context of sarcoma-like tumor processes, reinforcing its clinical relevance in addressing malignancies with complex genetic profiles [27,32]. However, the use of vincristine, like many chemotherapy agents, is frequently hindered by systemic toxicities and multidrug resistance mechanisms that compromise its overall success. Emerging research has sought to mitigate these challenges by exploring the integration of plant-derived polyphenols in cancer treatment paradigms. Methoxylated biphenolic ligands, such as nobiletin and tangeretin, have been identified as auxiliary agents with the ability to overcome resistance mechanisms linked to the overexpression of P-glycoprotein (P-gp). By inhibiting P-gp activity, these compounds enhance vincristine’s intracellular accumulation and cytotoxic potential in resistant cell lines, offering promising avenues for combination therapies [33,34]. Furthermore, bioinformatic tools, such as web-based docking platforms, have facilitated the identification of molecular targets and binding affinities, underscoring a precision-oncology approach that enhances the therapeutic index of vincristine [35].

In addition, natural phenolic and polyphenolic ligands have shown potential in modulating critical enzymatic and molecular pathways associated with tumor progression. For instance, quercetin has been reported to inhibit the phosphorylation of oncogenes such as the *Rous sarcoma virus*-transforming gene, while ligands like pinoresinol activate the ATM-p53 cascade, inducing apoptotic pathways and maintaining genetic stability. This emphasizes the role of natural compounds not only in the mitigation of resistance mechanisms, but also in diversifying the therapeutic landscape by targeting transcriptional and regulatory networks of cancer cells [36,37,38]. As a result, the combined application of vincristine and bioactive plant-derived compounds presents a dual benefit—enhancing clinical outcomes while reducing systemic toxicity and the risks associated with conventional single-agent chemotherapy. In the present study, toxicity-related parameters were obtained exclusively through in silico predictions. The value of 0.98 corresponds to the predicted probability of cytotoxic inactivity according to the ProTox-II model. Likewise, experimental data regarding mitochondrial membrane potential (MMP) and direct comparisons with vincristine were not generated in this work. Consequently, the discussion focuses on the predicted safety profile of pinoresinol and its docking-based affinity for the c-Myc–TBP–TAF1 complex, underscoring its relevance for future in vitro and in vivo studies [33].

These findings are consistent with the broader molecular effects ascribed to plant-derived ligands, such as pinoresinol. Mechanistically, the decrease in mitochondrial membrane potential (∆Ψm) indicates that mitochondrial dysfunction plays a pivotal role in apoptosis induction, a hallmark of the intrinsic apoptotic pathway. This dysfunction is often mediated by pro-apoptotic proteins such as Bax and Bak, which facilitate mitochondrial outer membrane permeabilization (MOMP) and promote the release of apoptotic factors, including cytochrome c, into the cytosol [37]. Similarly, the observed epithelial thinning may reflect the suppression of neoplastic cell expansion, driven by the activation of these critical regulatory pathways. For instance, these ligands are known to modulate key signaling molecules, such as p53 and c-Myc, thereby inducing G2/M cell cycle arrest, DNA fragmentation, and programmed cell death [39,40]. The therapeutic potential of pinoresinol and its structurally related compounds represents a remarkable advance in targeted cancer therapy. Pinoresinol has been demonstrated to selectively inhibit the proliferation of malignant cells while preserving the integrity of healthy tissues, which underscores its functional specificity as a candidate for safer and effective oncologic interventions. In silico analysis allows prediction; its ability to minimize cytotoxic effects on normal cells is supported by a predicted probability of 0.98 for cytotoxic inactivity, signifying its promising safety profile and favorable pharmacological characteristics. Expanding upon its mechanism of action, pinoresinol, a lignan-type ligand, mediates anticancer effects through plasma membrane perturbation without inducing hemolytic damage in human erythrocytes, offering a significant safety advantage over conventional chemotherapeutic agents [41,42]. Complementing the anticancer effects of pinoresinol, other bioactive compounds, such as hesperetin, exhibit synergistic therapeutic mechanisms, including the attenuation of mitochondrial potential and increased production of reactive oxygen species (ROS), which are key modulators of apoptotic signaling pathways [43]. These mechanisms align with the intrinsic apoptotic pathway, wherein mitochondrial dysfunction triggers cytochrome c release and initiates the executioner caspase cascade [37,39]. Such findings posit bioactive ligands as multi-targeted agents capable of simultaneously intervening in oncogenic pathways, such as MAPK and PI3K/Akt signaling, thereby enhancing both therapeutic efficacy and safety profiles [37,43].

Further elucidation of oncoprotein deregulation highlights the transformative impact of natural compounds on transcriptional regulators such as c-Myc and p53. Deregulated c-Myc facilitates replicative immortality and suppresses p53-mediated growth arrest, characteristics observed in over 50% of human cancers [15,37]. The 55% modulation of p53 observed in the study holds critical implications for cancer therapy, as p53 serves as the “guardian of the genome.” Its activation by bioactive ligands could induce cell cycle arrest at the G2/M phase or induce apoptosis in cells with irreparable genetic damage [39,43,44].

p53 is crucial, as it regulates the cell cycle and apoptosis, and plays a vital role in maintaining genetic stability. When genomic damage occurs, the damaged cells do not proceed through the cell proliferation cycle. Consequently, p53 levels increase rapidly, inducing apoptosis. In silico structural analysis utilizing the Research Collaboratory for Structural Bioinformatics PDB platform yielded the three-dimensional structure of the c-Myc-TBP-TAF1 macromolecular complex (PDB ID:6E16), which was subsequently visualized and prepared using Avogadro. Additionally, pinoresinol (C_20_H_22_O_6_, ID:73399) was examined on the PubChem and Protox-II platforms [32]. The compound exhibits an octanol/water partition coefficient of 3.19 and two hydrogen bond donors. These properties align closely with the reported literature [45,46], wherein the ^13^C-NMR spectrum exhibits ten signals for carbons; all of them represent two C atoms, differing slightly from epipinoresinol, which shares the same molecular weight but possesses a distinct stereochemical orientation of benzyl groups.

The investigation demonstrated that preparing the 6E16 complex and minimizing its energy yielded an optimal physical distance that suggested electrostatic interactions between the side chains. Energy minimization is a crucial computational step to ensure a stable and representative conformation of the macromolecular target, thereby reducing atomic strain and enhancing the accuracy of non-bonded interaction predictions [36]. Electrostatic forces, particularly hydrogen bonding, play a central role in ligand recognition and stabilization within protein pockets. The hydroxyl groups of phenolic ligands, known for their electron-donating properties, facilitate high-affinity anchoring that is essential for achieving molecular specificity during docking [39,42,47]. The computational setup employed UCSF Chimera^®^ 1.16, which utilizes the AMBER force field to model Van der Waals forces and Coulombic potentials effectively. AMBER ensures precise estimations of binding free energy, a fundamental metric in molecular docking studies. In silico methodologies have been successfully applied to identify natural inhibitors targeting hydrophobic or catalytic protein pockets, contributing to the modulation of oncogenic signaling pathways with high specificity [26,42,48,49].

The structural analysis revealed a balanced distribution of atomic properties across the macromolecular chains, integrating the characteristics of rings through logical operations. The aromaticity of the ligand, such as that of pinoresinol, plays a pivotal role during ligand docking, as its phenolic nucleus facilitates molecular interactions. Pinoresinol’s approximately symmetrical structure, which comprises twin aromatic rings, supports non-covalent interactions such as c-Myc and dispersion forces, thereby contributing to stability within the protein-ligand interface. The buried surface area, a key bioinformatic metric, also quantifies the extent of complementarity and interface integrity, highlighting the structural advantages of phenolic compounds within protein-ligand systems [31,44,50].

Molecular docking simulations were performed utilizing the AutoDock-Vina tool. The atomic force distribution indicators within the grid box were consistent with the predicted molecular interactions. After setting the exhaustiveness to 10, four atom types were identified: 1 = OA (oxygen-hydrogen bond acceptor); 2 = A (aromatic carbon); 3 = C (aliphatic carbon); and 4 = HD (hydrogen bond donor). The resulting binding modes fell within the spatial coordinates predicted by the AMBER model for the *x*, *y*, and *z* axes, displaying optimal binding affinity (kcal/mol). Regarding the evaluated biological activity of the identified ligands, the SeamDock platform effectively recognized the highest potential regions with the highest interaction potential. Furthermore, the SeamDock web server integrates various docking tools into a streamlined framework, facilitating both global and local ligand docking along with a hierarchical methodology for a more precise identification of molecular interaction sites. This dual docking strategy is particularly advantageous when examining molecular systems, such as transcription factors and oncoproteins, where binding pockets may remain partially undefined. Such specificity enables the effective targeting of “hotspots” for non-covalent interactions, an essential aspect for understanding and modulating protein-ligand dynamics.

Moreover, the comprehensive design of the SeamDock server allows the in-depth characterization of protein-ligand interfaces via advanced tools such as the NGL viewer. These analytical capabilities support the evaluation of hydrogen bonding, polar interactions, and conformational stability, which are critical factors in determining the functional efficacy of natural compounds. Drawing on these features, researchers can rationalize the biological activity of ligands and establish thermodynamic parameters, such as binding affinities (kcal/mol), as predictors of complex stability. The application of such integrated workflows has already demonstrated the recognition of potent natural inhibitors, such as molecules like piperlonguminine and berberine, whose binding affinities highlight their structural compatibility as drug candidates targeting critical enzymatic residues [35,44]. Likewise, the hierarchical ligand docking approach enabled by SeamDock facilitates the optimization of therapeutic potential, particularly for plant-derived secondary metabolites. These metabolites, known for their ability to establish stable non-covalent interactions, such as π-stacking and dispersion forces, are promising materials due to their high specificity and reduced systemic toxicity [39,42]. In conclusion, SeamDock plays an important role in the rational design of selective and efficacious therapeutics. This robust platform uniquely integrates diverse docking engines into a hierarchical framework, streamlining both global and local docking analyses to pinpoint optimal interaction sites effectively. Such advancements significantly enhance the characterization of protein-ligand interfaces by utilizing parameters including residue conservation and complementary physicochemical properties, which are instrumental in optimizing therapeutic design [35,51]. These interfaces often rely on the phenolic ligand nucleus, which serves as a key structural motif, facilitating critical non-covalent interactions like hydrogen bonding and dispersion forces with amino acid residues [39,47].

Ligand-binding site prediction remains a central focus in bioinformatics and computational studies. Advanced in silico approaches exploit sequential and structural information to identify binding sites, frequently supported by homology modeling and the recognition of hydrophilic hotspots, where residues, such as lysine or aspartic acid, act as principal binding anchors [35,52]. Through these computational tools, critical metrics such as buried surface area are calculated to quantify shape complementarity and to estimate binding free energy, providing researchers with predictive insights [42,53]. Additionally, these techniques facilitate the pre-screening of plant-derived secondary metabolites to identify molecules that selectively target biologically relevant proteins, such as oncogenic c-Myc or microbial enzymes. The application of such methodologies enhances therapeutic precision and safety [35,42,51].

Crucially, SeamDock exemplifies the value of integrating multidisciplinary strategies into therapeutic design. The tool employs computational, biochemical, and structural biology principles to offer a comprehensive framework for rational drug modeling. Such integration ensures compound identification with elevated specificity and efficient profiles, aligning with the precision medicine goals and the development of targeted therapies. Moreover, this synergistic approach enables researchers to navigate the complexities of molecular interactions with greater confidence and scientific rigor. In essence, SeamDock fosters a paradigm shift in drug discovery by bridging computational innovation with practical therapeutic applications. Its contribution highlights the transformative potential of interdisciplinary collaborations, consolidating the development of next-generation therapies capable of addressing complex medical challenges [25].

**Figure 9:**
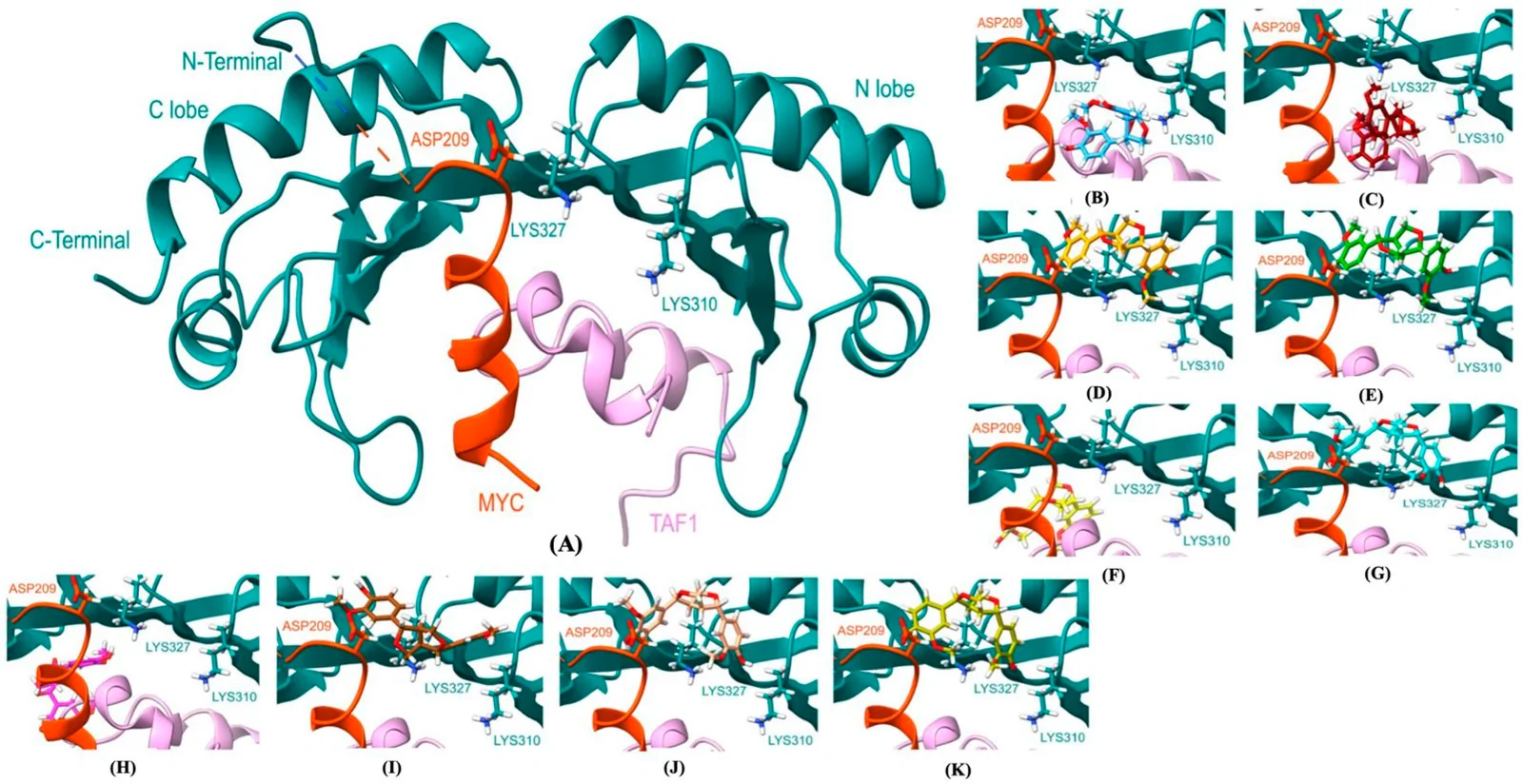
Positions and conformations of pinoresinol complex with c-Myc-TBP-TAF1: (**A**) Atomic coordinates of the experimentally determined c-Myc-TBP-TAF1 structure. The color code for the ligand positions and conformations (**B**–**K**), see Table 6 and Figure 6) is sky blue, maroon, orange, green, yellow, cyan, magenta, brown, salmon, and olive green for numbers 1 to 10, respectively.

As shown before, the ligands form interaction patterns on the receptor, according to the positions and conformations predicted by the molecular docking simulation, where residues LYS327, LYS310, and ASP209 developed interactions at more than four ligand interfaces. These patterns are repeated for the buried surface area of the interface for the same residues. However, residues AL326, GLN268, GLY208, LEU267, MET210, and PRO309 exhibit buried surface area at least over six interfaces but with a lower interface area (<10 Å), in proportion to the sum of the total interface area of LYS327, except for ASP331, GLY325, MET210, and THR266. All these residues exhibit the largest interface with respect to the ligands. However, all ligand positions and conformations are closer to residue LYS327 (Figure 9).

**Figure 10:**
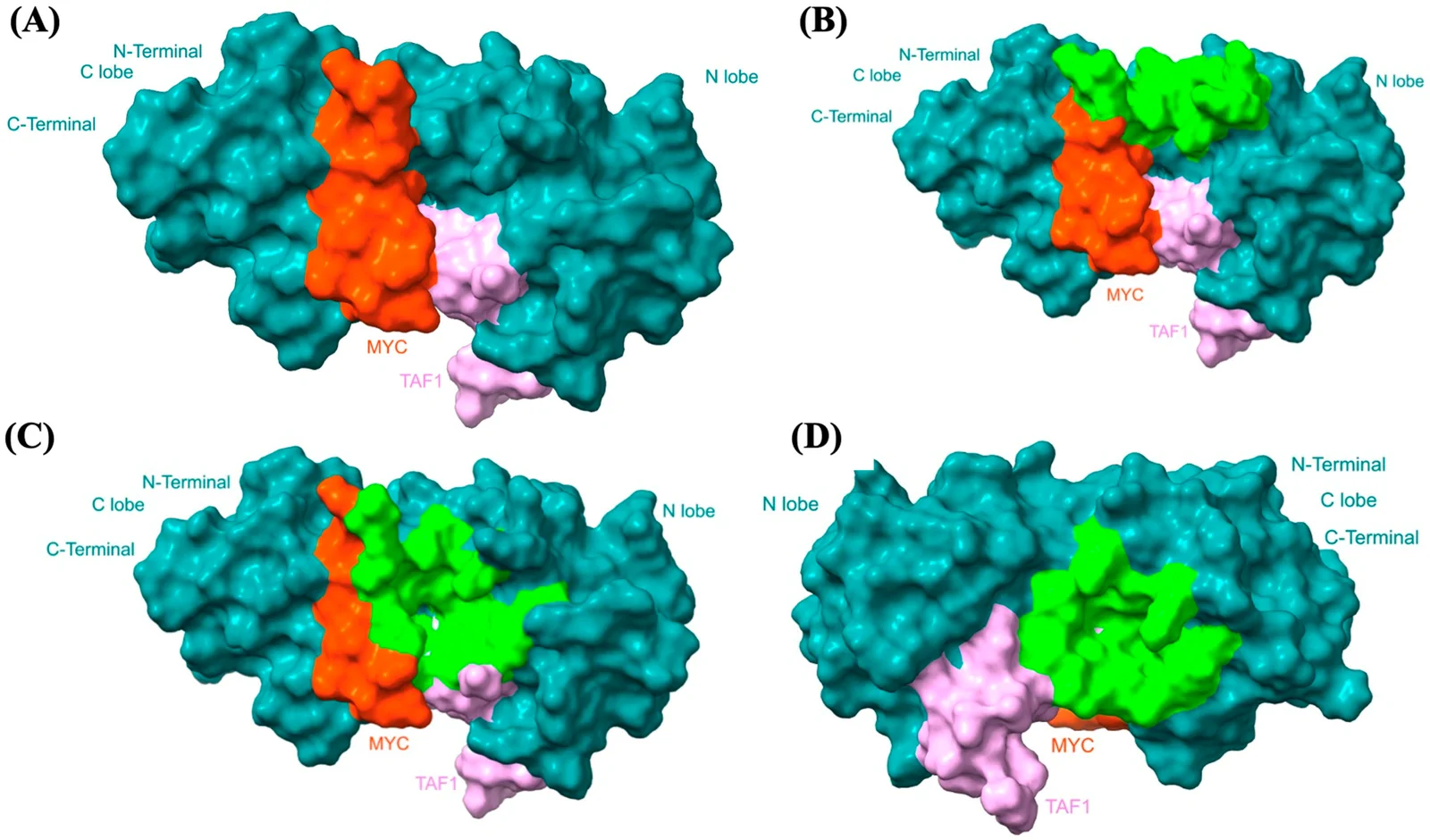
Solvent-excluded surface of amino acids (shown in light green) at the c-Myc-TBP-TAF1/Pinoresinol interface. “(**A**)” c-Myc-TBP-TAF1: Shows the baseline macromolecular complex, highlighting the c-Myc region in orange and the TAF1 segment in pink against the main protein structure in dark cyan. Lobes and terminal ends are labeled. “(**B**)” Pocket P_0 (P0): Highlights the interface pocket region mapped in light green near the upper boundary. “(**C**)” Pocket P_1 (P1): Demonstrates the extended light-green solvent-excluded surface area across the central binding interface adjacent to both the orange c-Myc and pink TAF1 regions. “(**D**)” Pocket P_2 (P2): Focuses on a distinct cavity profile mapped in light green, showing its spatial relation to the c-Myc and TAF1 contact points from a rotated or specific pocket-centric perspective. The protein surface structures and color-coded regions (cyan, orange c-Myc, pink TAF1, and light green binding pockets) are defined. The spatial distributions of pockets P0, P1, and P2 relative to the functional domains are clearly visible. Visual representation clearly conveys the distinct binding pockets involved in pinoresinol interaction at the c-Myc-TBP-TAF1 interface.

Moreover, the solvent-excluded area of the ligand interface residues can be identified in each pocket (P_0, P_1, and P_2) of the protein structure as plotted in Figure 10. The surfaces of the residues involved in the ligand interface in pockets P_0 and P_1 are in critical interaction regions of MYC. Unlike P_2, which lacks conventional hydrogen bonds and interfaces with the C lobe surface, and a minimal portion with TAF1, which also lacks hydrogen bonds. Because MYC oncoprotein interacts with the transcriptional machinery to promote gene expression, we hypothesize that the latter interaction site may not be a suitable target region for further study. Despite the results, we do not rule out any interaction site because how MYC interacts with the transcriptional machinery remains unclear.

## 5. Conclusions

This study provides an in silico evaluation of the biphenolic ligand pinoresinol as a possible modulator of the c-Myc–TBP–TAF1 transcriptional complex, a key regulatory node implicated in the development and progression of numerous cancers. Molecular docking and binding interface analyses reveal that pinoresinol shows stable and reproducible interactions within defined binding pockets (P_0_, P_1_, and P_2_) of the c-Myc–TBP–TAF1 complex, exhibiting moderate but favorable binding affinities, typical of small natural compounds.

Residues LYS327, LYS310, and ASP209, particularly LYS327, were consistently identified as interaction hotspots across multiple ligand conformations, highlighting them as promising molecular targets for disrupting c-Myc–associated transcriptional regulation. These findings support the hypothesis that pinoresinol may interfere with c-Myc function by targeting protein–protein interaction interfaces rather than catalytic sites, a strategy aligning with current approaches to address the historical “undruggability” of c-Myc.

In silico ADMET analysis predicted a generally favorable safety profile for pinoresinol. However, all toxicity-related results are strictly computational predictions and require experimental validation. Since no experimental cytotoxicity or mitochondrial dysfunction data were generated in this study, the results should be interpreted solely within the scope of predictive modeling.

Overall, this work highlights the utility of molecular docking, binding interface analysis, and chemometric approaches in identifying natural compounds capable of targeting oncogenic transcriptional complexes. Even though pinoresinol alone may not constitute a high-affinity inhibitor, its interaction pattern, structural symmetry, and predicted safety profile support its potential as a lead scaffold for further optimization. Future studies incorporating molecular dynamics simulations, structure-guided ligand refinement, and in vitro and in vivo validation will be essential to confirm the biological relevance and therapeutic potential of these findings.

## Figures and Tables

**Table 1:** Pinoresinol toxicity model (classification https://tox-new.charite.de/ (accessed on 28 January 2025)).

Classification	Target	Shorthand	Probability	Probability
Organ toxicity	Hepatotoxicity	Dili	Inactive	0.86
Toxiciendpointsnts	Carcinogenicity	carcino	Inactive	0.56
Toxicitendpointsts	Immunotoxicity	immuno	Active	0.89
Toxicity endpoints	Mutagenicity	mutagen	Inactive	0.77
Toxicity endpoints	Cytotoxicity	cyto	Inactive	0.98

**Table 2:** Physicochemical parameters of c-Myc-TBP-TAF1 and Pinoresinol.

Physical Parameters	c-Myc-TBP-TAF1	(+)-Pinoresinol
Atoms	3.410	48
Heavy atoms	1.678	26
Residues	226	n/a
Molecular weight (Da)	23,821.6	358.385
Geometry center	100.4668, 76.5442, −11.1885	4.793, −0.0001, −0.5716
Mass center	100.4737, 76.6372, −11.2166	4.793, −0.0001, −0.5306
Dimensions	57.424, 45.3512, 57.699	11.3859, 17.5253, 5.8348
Surface area (Å^2^)	11,079	621.5
Polar area (Å^2^)	4469.5	156.8
Apolar area (Å^2^)	6609.4	464.7
Volume (Å^3^)	21,757.5	325.1

**Table 3:** Biochemical toxicological characteristics of pinoresinol (https://tox-new.charite.de/ (accessed on 4 January 2025)).

Compound Name	Docking	Score (kcal/mol) Molecular Weight	Number of Hydrogen Bonds	Octanol/Water Partition Coefficient (logP)	Number of Hydrogen Bond Donors
Pinoresinol	0.82	358.39	2–5	3.19	2

**Table 4:** Number of points in the x and z dimensions, spaced in the center of the box (angstrom).

Dimension	x	y	z
Center	100.799	76.7381	−12.3822
Size	15.9295	41.2137	15.9866

**Table 5:** Accuracy of docking predictions.

Points	Model	R 95%	R-Adjusted	RMSE
Atom		0.48073	0.46342	2.0460
Nodes (repeats)		0.40224	0.38231	2.1952
Atoms and force	a∗exp(b∗x)	0.16201	0.15899	0.18795

**Table 7:** Identification of ligands with biological potential.

BS	LP	Hydrogen Bond	Weak Hydrogen Bond	Alkyl	Pi-Alkyl	Binding Affinity (kcal/mol)
P_0	3	3	3	3	2	−6.7
	4	1	2	2	1	−6.6
	6	0	1	1	2	−6.0
	8	2	0	2	3	−6.3
	9	4	1	2	2	−6.9
	10	5	1	1	3	−7.1
P_1	1	3	1	4	1	−6.4
	2	3	2	3	1	−6.8
P_2	5	0	4	1	0	−6.4
	7	2	0	0	0	−5.8

BS: Binding site; LP: Ligand pose.

## Data Availability

The original contributions presented in the study are included in the article; further inquiries can be directed to the corresponding author.

## Supplementary Materials

The following supporting information can be downloaded at: https://www.mdpi.com/article/10.3390/cimb48080833/s1.
