# Supplementary material for: Pinoresinol as a Potential c-Myc Complex Modulator: An In Silico Study
Source: Curr Issues Mol Biol. 2026 Aug 17;48(8):833. doi: 10.3390/cimb48080833 (PMC13511002; doi:10.3390/cimb48080833)
Supplement: Supplementary file 1 [file cimb-48-00833-s001.zip › cimb-4396752-supplementary.pdf]

Table S1. Chemometrics of non-covalent bonds at the interface of ligand 1 with c-Myc-TBP-TAF1

H-Donor	H-Aceptor	Pi-Orbitals	Bond type	Distance
A:THR324:HG1	B:MOL:O		Conventional Hydrogen Bond	2.76
A:LYS327:HZ1	B:MOL:O4		Conventional Hydrogen Bond	2.13
B:MOL:H	A:MET201:O		Conventional Hydrogen Bond	2.54
B:MOL:H8	A:THR324:OG1		Carbon Hydrogen Bond	2.92
B:MOL:C12	A:LEU30		Alkyl	4.58
B:MOL:C12	A:LEU205		Alkyl	4.09
B:MOL:C12	A:VAL361		Alkyl	3.84
B:MOL:C19	A:LEU205		Alkyl	5.16
	A:LEU205	B:MOL:R1	Pi-Alkyl	5.38

A: Protein chain corresponding to c-Myc-TBP-TAF1; MOL: Pinoresinol (+) ligand; R: Corresponds to aromatic rings 1 and 2 of the ligand.

Table S2. Chemometrics of non-covalent bonds at the interface of ligand 2 with c-Myc-TBP-TAF1

H-Donor	H-Aceptor	Pi-Orbitals	Bond type	Distance
A:LYS327:HZ1	B:MOL:O4		Conventional Hydrogen Bond	2.60
B:MOL:H	A:LEU30:O		Conventional Hydrogen Bond	1.86
B:MOL:H1	A:GLY207:O		Conventional Hydrogen Bond	2.55
B:MOL:H8	A:ASN269:OD1		Carbon Hydrogen Bond	2.64
B:MOL:H15	A:LEU30:O		Carbon Hydrogen Bond	2.61
B:MOL:C12	A:LEU30		Alkyl	4.73
B:MOL:C12	A:LEU205		Alkyl	4.74
B:MOL:C12	A:VAL361		Alkyl	4.42
	A:LEU205	B:MOL:R1	Pi-Alkyl	5.21

A: Protein chain corresponding to c-Myc-TBP-TAF1; MOL: Pinoresinol (+) ligand; R: Corresponds to aromatic rings 1 and 2 of the ligand.

Table S3. Chemometrics of non-covalent bonds at the interface of ligand 3 with c-Myc-TBP-TAF1

H-Donor	H-Aceptor	Pi-Orbitals	Bond type	Distance
A:LYS327:HZ3	B:MOL:O4		Conventional Hydrogen Bond	2.77
B:MOL:H	A:GLN268:OE1		Conventional Hydrogen Bond	2.35
B:MOL:H1	A:LYS310:O		Conventional Hydrogen Bond	2.09
A:ALA326:HA	B:MOL:O5		Carbon Hydrogen Bond	2.44
B:MOL:H15	A:LYS327:O		Carbon Hydrogen Bond	2.72
B:MOL:H22	A:LYS310:O		Carbon Hydrogen Bond	2.61
B:MOL:C12	A:LEU267		Alkyl	5.23
B:MOL:C12	A:LYS327		Alkyl	4.52
B:MOL:C19	A:LYS310		Alkyl	3.95
	A:LYS327	B:MOL:R1	Pi-Alkyl	4.36
	A:LYS327	B:MOL:R2	Pi-Alkyl	5.11

A: Protein chain corresponding to c-Myc-TBP-TAF1; MOL: Pinoresinol (+) ligand; R: Corresponds to aromatic rings 1 and 2 of the ligand.

Table S4. Chemometrics of non-covalent bonds at the interface of ligand 4 with c-Myc-TBP-TAF1

H-Donor	H-Aceptor	Pi-Orbitals	Bond type	Distance
B:MOL:H1	A:GLN268:OE1		Conventional Hydrogen Bond	2.21
A:ALA326:HA	B:MOL:O3		Carbon Hydrogen Bond	2.51
B:MOL:H21	A:LYS327:O		Carbon Hydrogen Bond	2.92
B:MOL:C12	A:LYS310		Alkyl	3.83
B:MOL:C19	A:LYS327		Alkyl	4.46
	A:LYS327	B:MOL:R2	Pi-Alkyl	4.27

A: Protein chain corresponding to c-Myc-TBP-TAF1; MOL: Pinoresinol (+) ligand; R: Corresponds to aromatic rings 1 and 2 of the ligand.

Table S5. Chemometrics of non-covalent bonds at the interface of ligand 5 with c-Myc-TBP-TAF1

**H-Donor**	**H-Aceptor**	**Pi-Orbitals**	**Bond type**	**Distance**
B:MOL:H15	A:GLU26:O		Carbon Hydrogen Bond	2.64
B:MOL:H16	B:MOL:O4		Carbon Hydrogen Bond	3.00
B:MOL:H21	A:PHE23:O		Carbon Hydrogen Bond	3.02
B:MOL:H21	A:GLY25:O		Carbon Hydrogen Bond	2.92
	A:VAL403	B:MOL:R1	Pi-Alkyl	4.46

A: Protein chain corresponding to c-Myc-TBP-TAF1; MOL: Pinoresinol (+) ligand; R: Corresponds to aromatic rings 1 and 2 of the ligand.

Table S6. Chemometrics of non-covalent bonds at the interface of ligand 6 with c-Myc-TBP-TAF1

**H-Donor**	**H-Aceptor**	**Pi-Orbitals**	**Bond type**	**Distance**
B:MOL:H14	A:ASP209:OD2		Carbon Hydrogen Bond	2.88
B:MOL:C19	A:LYS327		Alkyl	5.00
	A:LYS327	B:MOL:R1	Pi-Alkyl	5.14
	A:LYS327	B:MOL:R2	Pi-Alkyl	3.91

A: Protein chain corresponding to c-Myc-TBP-TAF1; MOL: Pinoresinol (+) ligand; R: Corresponds to aromatic rings 1 and 2 of the ligand.

Table S7. Chemometrics of non-covalent bonds at the interface of ligand 7 with c-Myc-TBP-TAF1

**H-Donor**	**H-Aceptor**	**Pi-Orbitals**	**Bond type**	**Distance**
A:SER29:HN	B:MOL:O5		Conventional Hydrogen Bond	2.17
A:GLN358:HE21	B:MOL:O4		Conventional Hydrogen Bond	2.82

A: Protein chain corresponding to c-Myc-TBP-TAF1; MOL: Pinoresinol (+) ligand; R: Corresponds to aromatic rings 1 and 2 of the

Table S8. Chemometrics of non-covalent bonds at the interface of ligand 8 with c-Myc-TBP-TAF1

**H-Donor**	**H-Aceptor**	**Pi-Orbitals**	**Bond type**	**Distance**
A:ASP209:HN	B:MOL:O		Conventional Hydrogen Bond	2.60
A:LYS310:HN	B:MOL:O4		Conventional Hydrogen Bond	2.17
B:MOL:C12	A:MET210		Alkyl	4.75
B:MOL:C19	A:LYS310		Alkyl	4.90
	A:LYS327	B:MOL:R1	Pi-Alkyl	4.90
	A:LYS310	B:MOL;R2	Pi-Alkyl	4.87
	A:LYS327	B:MOL:R2	Pi-Alkyl	4.86

A: Protein chain corresponding to c-Myc-TBP-TAF1; MOL: Pinoresinol (+) ligand; R: Corresponds to aromatic rings 1 and 2 of the ligand.

Table S9. Chemometrics of non-covalent bonds at the interface of ligand 9 with c-Myc-TBP-TAF1

H-Donor	H-Aceptor	Pi-Orbitals	Bond type	Distance
A:MET210:HN	B:MOL:O5		Conventional Hydrogen Bond	1.90
B:MOL:H	A:LYS310:O		Conventional Hydrogen Bond	2.41
B:MOL:H1	A:MET210:O		Conventional Hydrogen Bond	2.40
B:MOL:H1	A:GLN268:OE1		Conventional Hydrogen Bond	2.80
B:MOL:H20	A:ASP209:OD2		Carbon Hydrogen Bond	3.07
B:MOL:C12	A:LYS327		Alkyl	4.95
B:MOL:C19	A:MET210		Alkyl	4.68
	A:LYS327	B:MOL:R1	Pi-Alkyl	3.97
	A:LYS327	B:MOL:R2	Pi-Alkyl	5.06

A: Protein chain corresponding to c-Myc-TBP-TAF1; MOL: Pinoresinol (+) ligand; R: Corresponds to aromatic rings 1 and 2 of the ligand.

Table S10. Chemometrics of non-covalent bonds at the interface of ligand 10 with c-Myc-TBP-TAF1

H-Donor	H-Aceptor	Pi-Orbitals	Bond type	Distance
A:ASP209:HN	B:MOL:O4		Conventional Hydrogen Bond	2.07
A:LYS327:HZ2	B:MOL:O2		Conventional Hydrogen Bond	2.24
B:MOL:H	A:LYS310:O		Conventional Hydrogen Bond	2.28
B:MOL:H	A:GLY325:O		Conventional Hydrogen Bond	2.02
B:MOL:H1	A:MET210:O		Conventional Hydrogen Bond	2.91
A:GLY208:HA1	B:MOL:O4		Carbon Hydrogen Bond	2.77
B:MOL:C19	A:LYS327		Alkyl	4.87
	A:LYS310	B:MOL:R1	Pi-Alkyl	5.43
	A:LYS327	B:MOL:R1	Pi-Alkyl	4.38
	A:LYS327	B:MOL:R2	Pi-Alkyl	5.08

A: Protein chain corresponding to c-Myc-TBP-TAF1; MOL: Pinoresinol (+) ligand; R: Corresponds to aromatic rings 1 and 2 of the ligand.
